# Genetic mapping of a modifier locus affecting hypertension in soluble guanylate cyclase α_1_ deficient mice

**DOI:** 10.1186/1471-2210-11-S1-P14

**Published:** 2011-08-01

**Authors:** Emmanuel S Buys, Michael J Raher, Andrew Kirby, Sarah R Hayton, Laurel T Tainsh, Patrick Y Sips, Peter Brouckaert, Mark J Daly, Kenneth D Bloch

**Affiliations:** 1 Department of Anesthesia Critical Care and Pain Medicine, Massachusetts General Hospital, Boston, MA, USA; 2 Department of Medicine, Center for Human Genetic Research, Massachusetts General Hospital, Boston, MA, USA; 3 VIB Department of Molecular Biomedical Research, Ghent University, Ghent, Belgium

## Background

We previously reported that male mice deficient in the α_1_ subunit of the NO receptor soluble guanylate cyclase (sGCα_1_^-/-^ mice), an important nitric oxide (NO) receptor, are hypertensive [1]. The phenotype depends on the genetic background: sGCα_1_^-/-^ mice on a 129S6 (S6) background (sGCα_1_^-/-S6^) but not on a C57BL/6 (B6) background

(sGCα_1_^-/-B6^) develop hypertension [2]. These findings suggest that hypertension associated with sGCα_1_-deficiency is modulated by genetic factors. We aimed to identify modifier genes underlying the hypertension in sGCα_1_^-/-S6^ mice.

## Materials and methods

Mean arterial blood pressure (MAP) was measured invasively in 280 male F2 offspring from a sGCα_1_^-/-S6^ X sGCα_1_^-/-B6^ intercross (sGCα_1_^-/-F2^). All mice were genotyped with a genome-wide panel of 150 SNP markers for linkage analysis using the Sequenom MassArray system and MAPMAKER/QTL. Renin-1c and renin-2d genotyping was performed using gene-specific primers. Plasma renin activity (PRA) and aldosterone were measured in anesthetized male S6 wild-type (WT^S6^) and sGCα_1_^-/-S6^ mice by radioimmunoassay and enzyme-linked immunoassay, respectively. The renin angiotensin system (RAS) was blocked by treating mice with either the aldosterone receptor antagonist, Spironolactone (100 mg/kg/day, subcutaneous pellet for 7 days), or the renin inhibitor, Aliskiren (200mg/kg/day, by gavage for 10 days).

## Results

MAP in sGCα_1_^-/-F2^ mice varied between values observed in sGCα_1_^-/-S6^ and sGCα_1_^-/-B6^ mice. Linkage analysis identified a locus on chromosome 1 with a highly significant logarithm of odds (LOD) score of 6.1. This region is syntenic with previously identified hypertension-related QTLs in the human and rat genome and contains the gene coding for renin. Importantly, B6 mice have one renin gene (renin-1c), and S6 mice have two renin genes (renin-1d and renin-2). Presence of the renin-1d and renin-2 genes correlated significantly with elevated MAP in the F2 mice (P<0.0001). PRA was higher in sGCα_1_^-/-S6^ than in WT^S6^ mice (0.29±0.01 vs. 0.23±0.03 µg angiotensin 1/ml/hr, respectively, P<0.05). Similarly, plasma aldosterone levels were higher in sGCα_1_^-/-S6^ than in WT^S6^ mice (0.47±0.03 vs 0.34±0.03 ng/ml, respectively, P<0.05). Treatment with Spironolactone or Aliskiren normalized blood pressure in sGCα_1_^-/-S6^ (117±5 vs 146±2 mmHg, in Spironolactone and vehicle-treated mice, respectively, P<0.001, and 100±7 vs 148±4 mmHg, in Aliskiren and vehicle-treated mice, respectively, P<0.001).

## Conclusion

Together, these data identify renin as a possible genetic modifier of blood pressure in a setting of deficient NO-cGMP signaling. Furthermore, these findings highlight the importance of sGC in the regulation of the renin-angiotensin system (RAS) and suggest that sGC may be a therapeutic target in RAS-dependent hypertension.
